# Do Boat Traffic and Habitat Features Shape Habitat Use by Guiana Dolphins ( *Sotalia guianensis* ) in a Brazilian Estuary?

**DOI:** 10.1002/ece3.74004

**Published:** 2026-07-12

**Authors:** Alice Lima, Gastón Andrés Fernandez Giné, Khamila Tondinelli Souza Cruz, Niel Nascimento Teixeira, Gil Marcelo Reuss Strenzel, Yvonnick Le Pendu

**Affiliations:** ^1^ Graduate Program in Zoology Universidade Estadual de Santa Cruz Ilhéus Bahia Brazil; ^2^ Department of Biological Sciences Universidade Estadual de Santa Cruz Ilhéus Bahia Brazil; ^3^ Graduate Program in Tropical Aquatic Systems Universidade Estadual de Santa Cruz Ilhéus Bahia Brazil; ^4^ Department of Agricultural and Environmental Sciences Universidade Estadual de Santa Cruz Ilhéus Bahia Brazil

**Keywords:** anthropogenic impact, boat traffic, conservation management, estuarine ecosystems, habitat overlap

## Abstract

Increasing boat traffic in estuarine environments poses growing challenges for the conservation of coastal cetaceans, yet how these pressures interact with habitat features to shape spatial use remains poorly understood. This study investigated the influence of boat traffic and habitat features on the spatial use and habitat selection of Guiana dolphins ( *Sotalia guianensis* ) in Pontal Bay, a northeastern Brazilian estuary, using shore‐based observations and theodolite tracking of dolphin and boat trajectories collected between April 2015 and January 2016. Utilization distribution and habitat selection analyses revealed substantial overlap between dolphins and boats, particularly in deeper areas. At finer scales, however, dolphins preferentially used areas with lower boat traffic, greater depths, and proximity to fishing centers, while avoiding shallow zones and river edges. These patterns indicate a scale‐dependent trade‐off between access to foraging opportunities and exposure to anthropogenic disturbance. Our findings highlight the importance of integrating environmental and human‐related factors into estuarine management and support the need for measures that regulate vessel activity within key dolphin habitats.

## Graphical abstract

This study investigates how boat traffic and environmental factors shape the spatial distribution and habitat selection of Guiana dolphins in a northeastern Brazilian estuary. Although dolphins and boats overlapped broadly—particularly in deeper waters—dolphins showed a preference for areas with lower boat traffic, greater depths, and proximity to fishing centers, while avoiding shallow and river‐edge zones. These findings highlight a trade‐off between ecological benefits and human disturbance, emphasizing the need for estuarine management strategies that balance conservation and human activities.

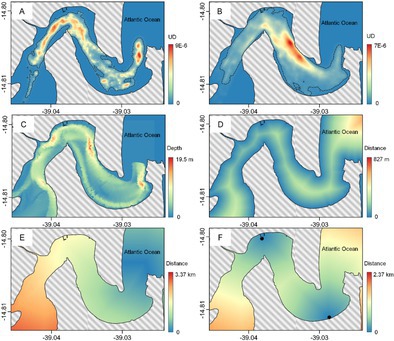

## 1 Introduction

Estuarine waters are among the most productive coastal environments on the planet. They provide crucial resources and conditions for several marine mammal species, but they are also the most heavily impacted by human activities (Mann 2000). In these environments, marine mammals are exposed to multiple anthropogenic pressures—from altered flow regimes and nutrient inputs to noise and boat traffic—which can degrade habitat quality. These impacts can alter the behavior of marine mammals and even affect their long‐term presence in an area (Erbe et al. 2019; Bridge et al. 2023; Silva et al. 2024). In addition to direct disturbance, human activities can also modify the distribution and availability of prey through processes such as fishing, habitat alteration, and nutrient inputs (e.g., Lotze et al. 2006; Halpern et al. 2008), thereby indirectly shaping marine mammal habitat use (Nabe‐Nielsen et al. 2018). Boat traffic stands out as a major source of disturbance for marine mammals in coastal ecosystems where human activity is intense and persistent (Halpern et al. 2008; de Almeida et al. 2024). Understanding the influence of both natural and anthropogenic factors on the spatial behavior of marine mammals in estuarine ecosystems offers critical insights into how they respond to environmental heterogeneity and human‐induced pressures. Such knowledge is useful for developing conservation and management strategies (Villero et al. 2017).

In many coastal systems, marine mammals and human activities tend to concentrate in areas defined by similar environmental features, such as depth, accessibility, and productivity hotspots. This spatial convergence increases the likelihood of interactions and potential conflicts, particularly in regions with intense vessel traffic (Nowacek et al. 2007; Jensen et al. 2009; Pirotta et al. 2015; La Manna et al. 2023). Such effects are especially pronounced for small coastal cetaceans, which often exhibit strong site fidelity and rely on restricted habitats (Bearzi et al. 2008; Batista et al. 2014). In this context, habitat use can be driven by resource acquisition, exposure to anthropogenic disturbance, or the balance between these factors. Understanding how animals respond to these overlapping spatial constraints is therefore critical not only for species‐specific conservation but also for effective spatial management in estuarine systems (Maxwell et al. 2015).

The Guiana dolphin ( *Sotalia guianensis* ) is a small coastal cetacean endemic to the tropical western Atlantic, occurring primarily in shallow coastal and estuarine waters from Central to South America (Flores et al. 2018; Secchi et al. 2018; de Jesus Lobo et al. 2021). The species commonly exhibits site fidelity within these ecosystems (Azevedo et al. 2004; Rossi‐santos et al. 2007), foraging primarily on fish, with cephalopods also reported in its diet (Santos et al. 2002).

The species is listed as “Near Threatened” on the IUCN Red List (Secchi et al. 2018) and “Vulnerable” to extinction in Brazil (BRASIL 2022). It is particularly susceptible to habitat degradation, pollution, bycatch, and boat traffic due to its strong dependence on coastal and estuarine systems (da Silva and Best 1996; Flores et al. 2018; de Jesus Lobo et al. 2021). Suitable habitat is expected to decline in the coming decades due to climate change (Tardin et al. 2025), while increased maritime traffic exacerbates risks such as collisions, acoustic disturbances, and changes in prey availability. These pressures highlight the need for robust assessments of the factors affecting population viability and for targeted management actions (Azevedo et al. 2017; de Almeida et al. 2024).

Previous research highlights several environmental variables that shape the habitat preferences of Guiana dolphins, including tidal state (Paitach et al. 2017), depth (Geise et al. 1999; Ferro de Godoy et al. 2015), water temperature (Borobia et al. 1991), slope, distance from shore (Wedekin et al. 2010), and prey distribution (Daura‐Jorge et al. 2007). Water depth preferences vary across study areas, and the dolphins are often found in shallow waters near sandbanks and the coastline (Azevedo et al. 2007). These variables influence dolphin distribution largely through their effects on prey availability (David 2002; MacLeod 2009) and can also interact with predator–prey dynamics (Redfern et al. 2006; Wedekin et al. 2007; Tardin et al. 2020). In southern Brazil, Guiana dolphins favor areas near the bay's mouth over channels and more remote spots (Wedekin et al. 2010), a preference likely due to increased foraging efficiency—a behavior also seen in bottlenose dolphins (Mendes et al. 2002). The proximity of fishing and aquaculture areas may also influence the spatial distribution of Guiana dolphins, which often remain near these operations, likely due to increased prey availability (Tardin et al. 2020; de Meirelles et al. 2023). This may occur through mechanisms such as fish aggregation around artificial structures and the enhanced availability of discards and bycatch (Fertl 1997; Díaz López and Methion 2017; Diáz López 2019; Methion and Díaz López 2019). Conversely, dolphins may avoid such areas because of fishing activity, limited access to foraging areas and vessel traffic pressures (Bearzi 2002; Markowitz et al. 2004; Read et al. 2006; Ribeiro et al. 2007; Pearson et al. 2012; Bordin et al. 2022). These contrasting effects highlight the dual role that human activities may have in shaping dolphin habitat use, simultaneously influencing resource availability and exposure to disturbance.

Although some studies have investigated the behavioral effects of boat traffic on Guiana dolphins (Carrera et al. 2008; Marega et al. 2018), few have examined the spatial overlap of small cetaceans with boats (La Manna et al. 2023), and this interaction has not yet been studied in an estuarine environment. Encounters with boats generally elicit neutral responses from Guiana dolphins (Araújo et al. 2008), although short‐term behavioral changes, including avoidance, have been observed in some contexts (Carrera et al. 2008; Tosi and Ferreira 2009; Marega et al. 2018). Beyond these immediate responses, however, dolphins may also adjust their longer‐term habitat use by avoiding areas that are consistently or intensively used by boats, for example after repeated exposure to disturbance or collisions. With increasing human activity in estuaries, understanding how dolphins select and use space in relation to both environmental conditions and anthropogenic stressors is critical to assessing their vulnerability in these ecosystems. Identifying potential conflict zones in estuarine environments can effectively inform conservation strategies and mitigation efforts.

Furthermore, habitat selection is inherently scale‐dependent, as the environmental and spatial features influencing animal distribution may vary across different spatial extents (Johnson 1980). At broader scales (second‐order selection, *sensu* Johnson, 1980), individuals select the general areas or home ranges they occupy, whereas at finer scales (third‐order selection) they differ in how intensively they use locations within those areas. The same environmental factor, such as anthropogenic disturbance, may have little influence on where home ranges are established but strongly affect the intensity of use within them (Johnson 1980; Lipsey et al. 2017). Adopting a multiscale approach is therefore essential to identify critical habitats, understand the underlying habitat selection, and inform effective conservation and management strategies (Boyd et al. 2008; Lipsey et al. 2017).

Here, we use the Guiana dolphin as a model species to investigate how habitat features and boat traffic jointly shape habitat use in a tropical estuary. Our focus is on spatial patterns of habitat selection in relation to the overall intensity of boat traffic, rather than short‐term behavioral responses to the immediate presence of boats. We quantified the spatial overlap between dolphins and boats and assessed how boat traffic intensity, water depth, and distance to the river edge, ocean, and fishing centers influence dolphin habitat use and selection at two spatial scales: the broader study area (second‐order selection) and within the dolphins' area of use, reflecting variation in intensity of use (third‐order selection). We hypothesize that, at both scales, dolphins would prefer deeper areas and exhibit lower relative use of areas with higher boat traffic intensity and proximity to riverbanks, due to anthropogenic disturbances. Additionally, we expect dolphins to favor areas close to the ocean (i.e., the estuary mouth) and fishing centers, where prey availability is likely to be high.

## 2 Materials and Methods

### 2.1 Study Area and Environmental Context

**FIGURE 1:**
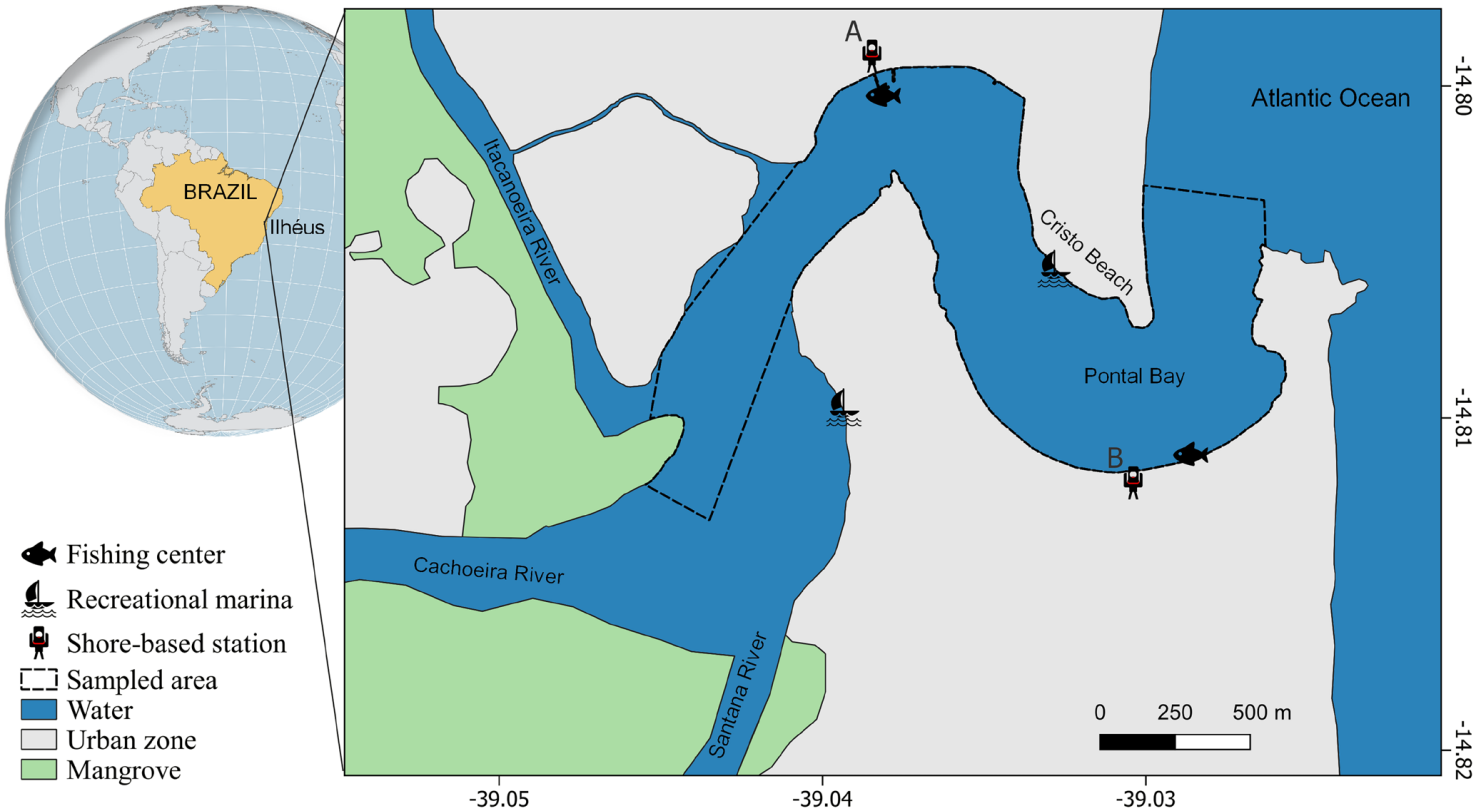
Study area and observation points in Pontal Bay, Ilhéus, Brazil. The dashed line indicates the sampled area. Observation points: Point A (−39.038102; −14.799506; elevation 6.56 m) and B (−39.030502; −14.811948; elevation 17.95 m).

The study was conducted in the lower Cachoeira River estuary, also known as Pontal Bay, located in Ilhéus, Bahia, Brazil (39°00′ to 39°03′ W; 14°47′ to 14°50′ S). This estuary is formed by the confluence of three rivers and flows into the Atlantic Ocean (Figure 1). The depth of Pontal Bay ranges from 0.6 to 19.5 m and its width ranges from 200 to 540 m (Matos 2017). Boats depart from three docks and a beach: motorboats used by artisanal fishers leave mainly from a fishing terminal and the facilities of a fishing community; small motorboats and jet‐skis use a private marina, while kayaks and small sailboats are rented at Cristo beach. Artisanal fisheries in the study area are conducted at small spatial scales in coastal and estuarine environments, typically involving small vessels operating close to shore and using a variety of fishing gears such as gillnets and handlines (FAO 2011). Pontal Bay is surrounded by urban areas, where boat wrecks, rubble dumping, and domestic sewage are common (da Silva et al. 2019). Mangrove vegetation is found only on the western side of the estuary (Figure 1).

### 2.2 Data Collection

We recorded the trajectories of Guiana dolphin groups and boats from two shore‐based stations (Figure 1A,B), which offered a panoramic view of the study area. The monitored area corresponded to the region delimited by the dashed line shown in Figure 1. Monitoring took place 3 days a week from April 2015 to January 2016. Each sampling day consisted of two observation sessions, chosen from three time periods (7:00–10:00, 10:30–13:30, and 14:00–17:00). The schedule was balanced to ensure each time period was covered by eight sessions per month. The specific monitoring station, day, and time period were assigned each week using a pseudorandom sequence. This schedule resulted in 24 sessions per month, for a total of 542.57 h of data collection.

#### 2.2.1 Dolphin Monitoring

During each monitoring session, we scanned the area for dolphins using the naked eye and binoculars (Ocean Xtreme 7x50WP, Lugan). A group was defined as any individual within a 100 m radius of each other (Irvine et al. 1981). During tracking, group cohesion was monitored continuously. When a fusion event occurred (i.e., additional individuals joined the focal group within 100 m), these individuals were incorporated into the monitored group. When a fission event occurred (i.e., individuals moved beyond 100 m from the focal group), we continued tracking the original focal subgroup without splitting the trajectory.

Once a group was sighted, we determined its initial and subsequent locations (geographical coordinates) via triangulation using a digital theodolite (Leica Model T110) from two reference positions with known coordinates and elevation. Group locations (and their associated times) were recorded at the shortest possible time intervals (typically around 10 s) while the group remained visible. Each location corresponded to the position of the individual closest to the center of the group at the time of surfacing. Trajectories were constructed by connecting successive locations separated by a maximum of 4 min, resulting in 1204 locations and 89 trajectories.

#### 2.2.2 Boat Monitoring

Boat trajectories were recorded during periods when no dolphin groups were present in the study area, allowing uninterrupted tracking of boat movements. Like dolphin tracking, the boat's geographic coordinates and corresponding timestamps were recorded at short intervals. Each boat, whether motorized or not, was monitored for up to 4 min or until it left the study area or stopped moving. This sampling yielded 4365 boat trajectories, comprising 39,541 locations. These data were used to derive boat utilization distributions (UDboats), representing spatial patterns of boat traffic intensity.

#### 2.2.3 Habitat Features and Anthropogenic Variables

We generated 1‐m resolution, georeferenced digital layers (rasters) representing habitat features and anthropogenic influence in Pontal Bay. These included depth (DEP), river edge distance (RED), and ocean distance (OD), which describe natural spatial gradients, as well as distance to fishing centers (FCD), representing the distance to fishing‐related human activities. Exploratory analyses did not reveal consistent temporal changes in dolphin spatial use during our study period; thus, temporal variables were not included in our analysis.

The depth layer was derived from a bathymetric survey conducted in June 2016, which consisted of 26,624 sampling points and was interpolated using Empirical Bayesian Kriging (Matos 2017). The distance‐based rasters (RED, OD, and FCD) were calculated using the gridDistance function from the raster package (Hijmans 2023), representing the minimum Euclidean distance from each cell to the nearest target, measured along the river's course. RED was calculated from the high tide line, OD was measured from the estuary's outlet to the ocean, and FCD was based on the locations of key fishing centers (the fishing terminal and the fishermen's association facilities).

### 2.3 Data Analysis

#### 2.3.1 Spatial Use and Overlap Analysis of Dolphins and Boats

First, we analyzed the spatial use of dolphins and boats using the utilization distribution (UD), a probability density function that quantifies the likelihood of use across geographical space. The UD is represented on a spatial grid where each cell receives a value proportional to the probability of use, so that higher values at a given location indicate a higher likelihood that dolphins and boats use that space. We estimated the utilization distribution for dolphins (UDdolphins) and boats (UDboats) from their trajectories using the biased random bridge kernel method (BRBK/MKDE) with a 1‐m raster resolution (Benhamou and Cornélis 2010), implemented with the BRB function from the adehabitatHR package (Calenge 2024).

We set the smoothing parameter hmini to 5 m for both dolphin and boat locations, reflecting location uncertainty. The diffusion coefficient (D), which describes how quickly an individual's position diffuses through space under a diffusion process (Benhamou 2011), was estimated by maximum likelihood using the “BRB.likD” function from the adehabitatHR package (Benhamou and Cornélis 2010). Trajectories were considered distinct when the time interval between successive locations exceeded 4 min (Tmax = 240 s), a value based on the temporal distribution of the tracking data, above which time gaps and step lengths became excessively large, thereby avoiding unrealistic interpolations over long steps between successive locations (Calenge 2024). Additionally, successive locations were considered distinct when separated by more than 0.1 m (l min = 0.1 m). The tau parameter, which controls the time lag between interpolated locations (Calenge 2024), was set to 6 s, resulting in approximately 3–5 interpolation points between successive locations, given median time intervals of 21 s for boats and 31 s for dolphins.

Based on the estimated UDs, we used the 95% and 50% kernel probability density thresholds to delineate the area of use (BRBK95%) and core area (BRBK50%) for dolphins and boats, and to quantify the overlap between their areas of use.

#### 2.3.2 Habitat Use and Selection

We conducted an exploratory multivariate analysis using the canonical Outlying Mean Index (OMI) (Doledec et al. 2000) to examine differences in dolphin and boat environmental space use within the multidimensional environmental space of Pontal Bay, defined by DEP, RED, OD and FCD. For this analysis, we extracted the values of these variables for each dolphin group and boat location (representing the used environmental space) and for each cell in the environmental layers (representing the available environmental space). Multivariate analyses were then run using the *canomi* function from the *adehabitatHS* package (Calenge 2024) to assess how the environmental space occupied by dolphins and boats deviated from the centroid (mean) of the available multivariate environmental space (Doledec et al. 2000). This analysis provided a preliminary characterization of the environmental gradients occupied by each and highlighted the differences between them.

Next, we analyzed dolphin habitat selection at two spatial scales: the study area (Pontal Bay) and the area of use scale. These correspond to the second‐ and third‐order habitat selection proposed by Johnson (1980). Second‐order selection assessed the environmental characteristics that dolphins selected to establish their areas of use within Pontal Bay. Third‐order selection, in contrast, focused on the characteristics chosen that shaped the distribution of use within their respective areas of use.

At the Pontal Bay scale (second‐order selection), we used GIS to randomly place 196 points (locations) within the sampled area, ensuring a minimum distance of 60 m between points. Points that fell within the dolphins' area of use (BRBK 95%) were classified as “used” (1), while those outside this area were considered “unused” (0). For each point, we then extracted the values for DEP, RED, OD, FCD, and UDboats and confirmed low collinearity among these variables (Pearson's *r* < 0.70). To assess the influence of these explanatory variables, on the second‐order habitat selection, we fitted Generalized Linear Models (GLMs) with a binomial distribution and logit link function using the *glm* function from the *stats* R package (R Core Team 2024). We then used the *dredge* function from the *MuMIn* package (Bartoń 2023) to evaluate and compare a full set of models, including all possible variable combinations and the null model (32 models total). The ‘best’ candidate models were selected based on the corrected Akaike Information Criterion (AICc). Models with a ∆AICc value of less than 2 were considered equally plausible. We selected the most parsimonious model among those with the lowest AICc values (Burnham and Anderson 2002) to identify which explanatory variables influenced dolphin habitat selection at this scale. The direction of these effects was inferred from the sign of the estimated coefficients.

At the scale of the area of use (third‐order selection), we evaluated how habitat features and anthropogenic variables influenced the utilization distribution (UD) of dolphins within their used area in Pontal Bay. For each dolphin location, we extracted the values of the response variable (UDdolphins) and the explanatory variables (DEP, RED, OD, FCD, and UDboats). Next, we fitted linear mixed‐effects models (LMMs) using the *lmer* function from the *lme4* package (Bates et al. 2015), specifying each trajectory as a random variable. We accounted for spatial autocorrelation among successive locations using the *corCAR1* function from the *nlme* package in R (Pinheiro and Bates 2000, 2023), including the continuous time elapsed since the start of the experiment as a covariate in the correlation structure. A preliminary analysis based on AICc indicated that the *corCAR1* function provided a better fit than the *corARMA* and *corAR1* functions. Similar to the second‐order selection analysis, we used the *dredge* function to test and compare all possible combinations of variables against the null model. We then selected the best‐fitting model based on AICc to identify the explanatory variables that affected dolphin habitat selection at this scale.

We used the same analytical procedure to evaluate the influence of explanatory variables (DEP, RED, OD and FCD) on boat space use at second‐ and third‐order scales. Boat trajectories reflect decisions of human operators in response to environmental and anthropogenic features. Accordingly, we modeled boat spatial patterns using the same statistical framework employed for dolphin habitat selection but interpret the outputs as patterns of boat distribution within the environmental space, rather than as habitat use or selection. Although boats were initially classified by type (e.g., motorized vs. non‐motorized), strong collinearity between overall boat utilization distribution (UDboats) and individual boat categories (Pearson's *r* > 0.7–0.8) led us to retain a single composite variable (UDboats) in data analysis to avoid redundancy and improve model interpretability. All analyses were performed in R (R Core Team 2024), and maps were generated using *QGIS* 3.36.3 (QGIS.org 2026).

Ethical review and approval were not required for this animal study, as all surveys were land‐based and involved no physical sampling or disturbance of the dolphins.

## 3 Results

### 3.1 Spatial Use and Overlap

**FIGURE 2:**
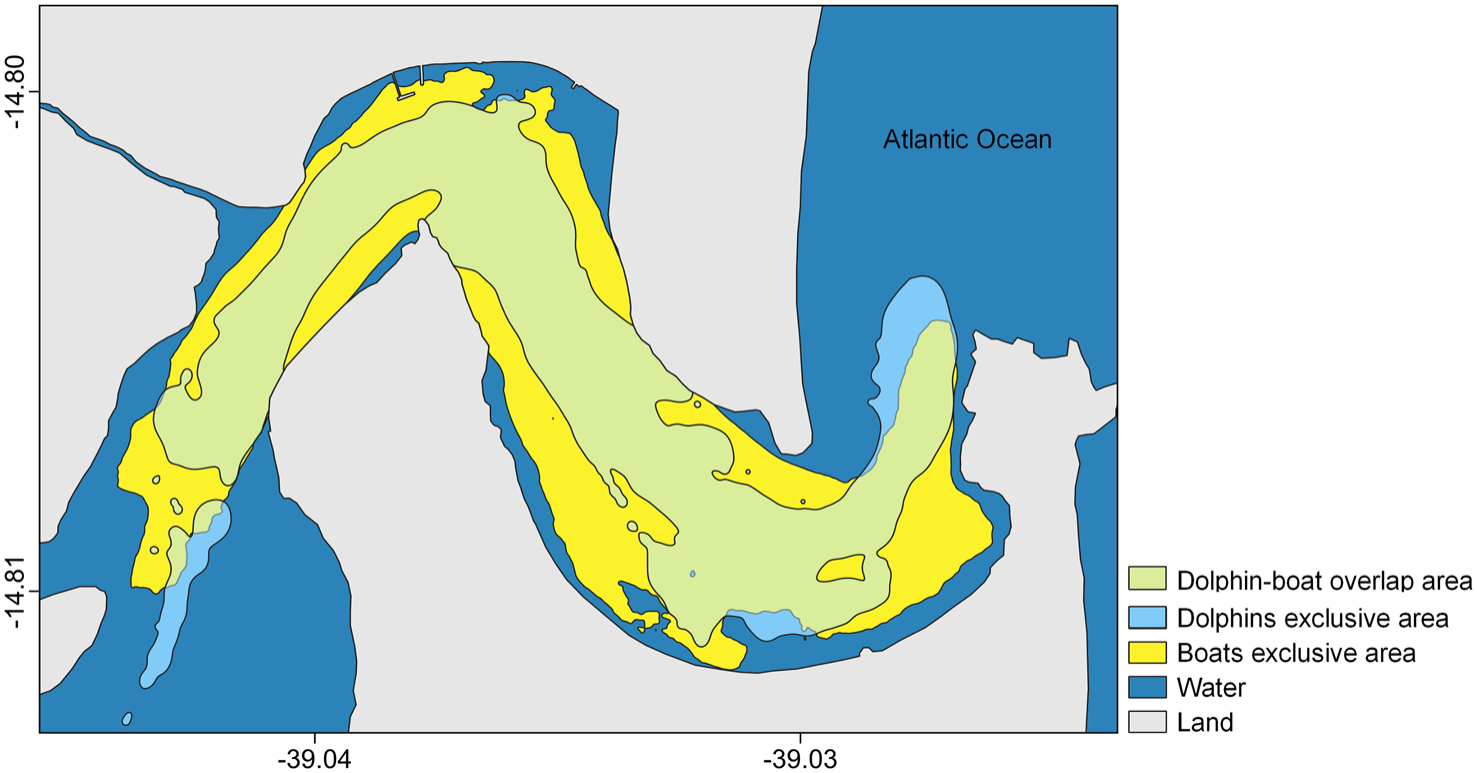
Total area of use by Guiana dolphins and boats, and their area of overlap, in Pontal Bay from April 2015 to January 2016. Spatial use was estimated using the Biased Random Bridge kernel (BRBK) method, and the area of use was defined by the 95% utilization distribution (UD) contour.

The Guiana dolphins' area of use (BRBK95%) was 63.38 ha, while the boats' was 94.59 ha, accounting for 44.46% and 66.36% of the sampled area, respectively. There was a 90.12% overlap between the areas used by boats and by dolphins (Figure 2).

### 3.2 Habitat Use and Selection

**FIGURE 3:**
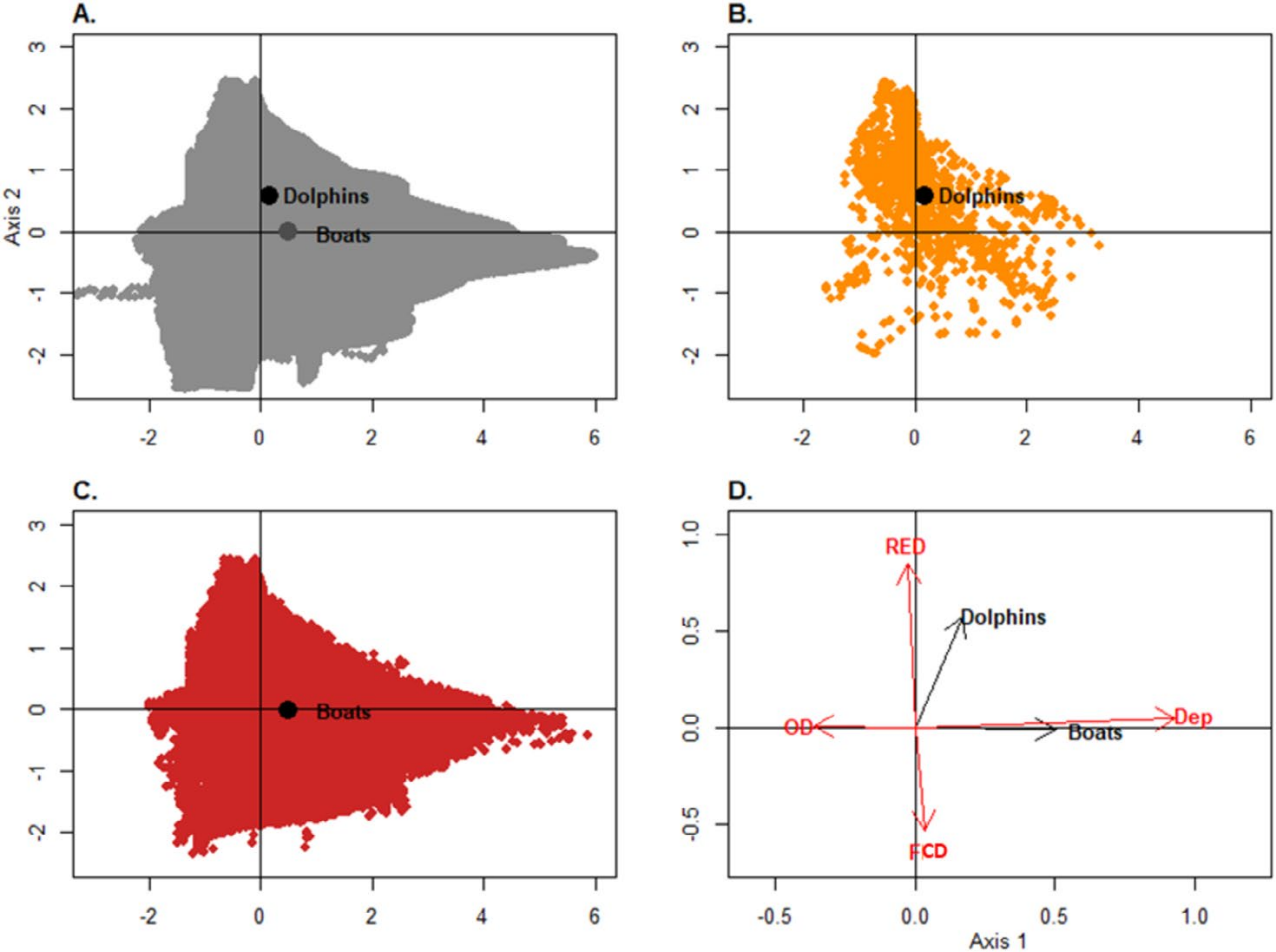
Canonical outlying mean index (OMI) analysis of the environmental space used by dolphins and boats. (A) The total environmental space available in the study area, where the intercept between axes 1 and 2 corresponds to the centroid of the available space, shown with the centroids of the environmental space used by dolphins and boats. (B) Environmental space used by Guiana dolphins ( *Sotalia guianensis* ) and (C) by boats, each with its centroid along axes 1 and 2. (D) Environmental variable vectors superimposed on the marginality vectors for dolphins and boats, indicating how each variable contributes to the deviation between available and used spaces. Variables are habitat features: Distance from the river edge (RED), water depth (DEP), and distance from the estuary mouth/ocean (OD); and the anthropogenic variable: Distance from fishing centers (FCD).

**FIGURE 4:**
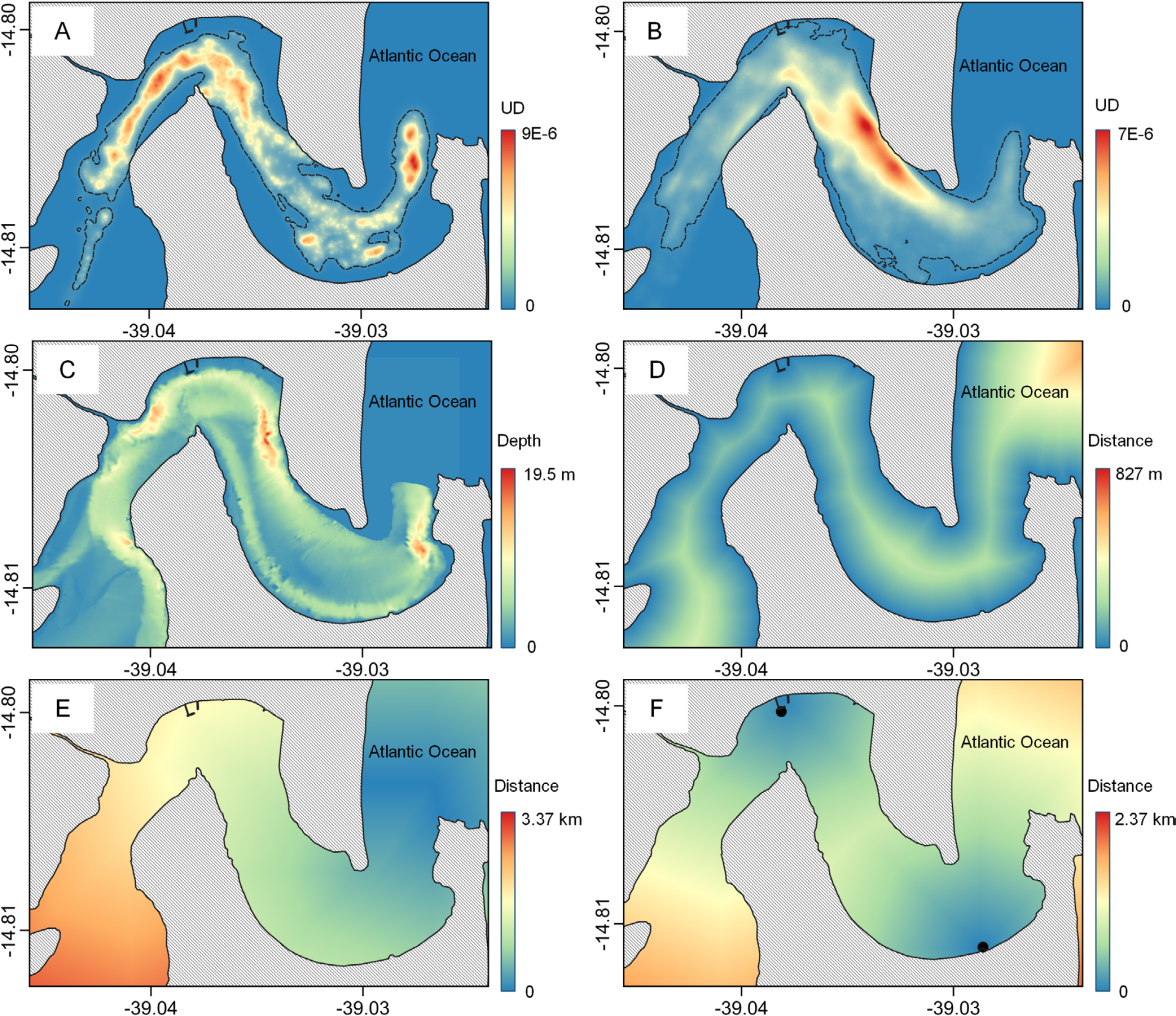
Spatial use of Guiana dolphins ( *Sotalia guianensis* ) and variables in the study area. (A) Dolphin utilization distribution (UD) estimated using the Biased Random Bridge Kernel method; dashed contour lines indicate the area of use based on the 95% UD contour. (B) UD and area of use for boats, estimated using the same method. In (A) and (B), the color gradient (blue to red) represents increasing use intensity. (C) Bathymetry (DEP), where darker shades indicate deeper waters. (D) Distance from the river edge (RED). (E) Distance from the estuary mouth/ocean (OD). (F) Distance from fishing centers (FCD), where black dots mark their locations. For (D–F), the color gradient (blue to red) indicates increasing distances.

In the Canonical OMI Analysis (Figure 3), the first two axes explained 99.99% of the data variation, with the first and second axes accounting for 96.13% and 3.86%, respectively. The environmental space used by dolphins in Pontal Bay was narrower than that occupied by boats (Figure 3), as dolphins tended to occur in areas at greater depths (DEP) and farther from the river edges than boats (Figures 3B,D and 4). In contrast, boats occupied almost the entire environmental and geographical space of Pontal Bay (Figures 3C and 4), with a higher occurrence in the deeper areas of the bay (Figures 3D and 4).

Within Pontal Bay (second‐order selection), the best‐fitting model indicated that UDboats, DEP, RED, and FCD influenced dolphin habitat selection (Table 1a). Dolphin area of use was positively influenced by UDboats, DEP, and RED, while FCD had a negative effect. Boat spatial distribution showed a similar pattern, with higher occurrence in deeper waters, areas farther from the river edges, and closer to fishing centers (Table 2). Distance to the ocean (OD) had no effect on area selection for either dolphins or boats.

**TABLE 1:** AICc‐based model selection for variables influencing habitat use by Guiana dolphins ( *Sotalia guianensis* ) in Pontal Bay.

Model rank	Candidate models^a^	Df	AICc	Δi	ωi
(a) Area of use in the estuary (second‐order habitat selection)					
1	**DEP** ^**+**^ + **RED** ^**+**^ + **FCD** ^**−**^ + **UDboats** ^**+**^	5	174.5	0	0.595
2	DEP^+^ + RED^+^ + OD^−^ + FCD^−^ + UDboats^+^	6	176.6	2.12	0.206
3	RED^+^ + FCD^−^ + UDboats^+^	4	178.7	4.24	0.071
(b) Utilization distribution within the area of use (third‐order habitat selection)					
1	**DEP** ^**+**^ + **RED** ^**+**^ + **FCD** ^**−**^ + **UDboats** ^**−**^	8	−29762.6	0	0.555
2	DEP^+^ + RED^+^ + OD^−^ + FCD^−^ + UDboats^−^	9	−29760.8	1.75	0.232
3	DEP^+^ + FCD^−^ + UDboats^−^	7	−29759.8	2.77	0.139
4	DEP^+^ + OD^−^ + FCD^−^ + UDboats^−^	8	−29758.4	4.17	0.069

*Note:* Model selection results based on corrected Akaike Information Criterion (AICc) for the variables that influence (a) The dolphins' overall area of use within the estuary and (b) Their utilization distribution (UD) within their local area of use. Predictor variables included: Bathymetry (DEP), distance from the shore (RED), distance from the estuary entrance (OD), distance from fishing centers (FCD), and boat utilization distribution (UDboats). The table also shows the number of degrees of freedom (df), differences in AICc (ΔAICc), and Akaike weights (ωi).

^a^ This table shows the top‐ranked models from a set of 32 models built using all possible combinations of explanatory variables, in addition to the null model. Models with ΔAICc < 2 are considered equally plausible in explaining the response variables. Bold indicates the most parsimonious model among them (i.e., the best‐supported model with the lowest AICc). Superscripts indicate positive or negative relationships for each variable.

**TABLE 2:** AICc‐based model selection for variables influencing the area of use by boats in Pontal Bay.

Model rank	Candidate models^a^	Df	AICc	Δi	ωi
(a) Area of use in the estuary (second‐order habitat selection)					
1	DEP^+^ + RED^+^ + OD^+^ + FCD^−^	5	169.8	0	0.685
2	**DEP** ^**+**^ + **RED** ^**+**^ + **FCD** ^**−**^	4	171.4	1.56	0.314
3	DEP^+^ + RED^+^ + OD^−^	4	184.0	14.17	0.001
(b) Utilization distribution within the area of use (third‐order habitat selection)					
1	**DEP** ^**+**^ + **RED** ^**−**^ + **FCD** ^**+**^	7	−32541.2	0	0.648
2	DEP^+^ + RED^−^ + OD^−^ + FCD^+^	8	−32539.6	1.64	0.286
3	DEP^+^ + RED^−^	6	−32536.0	5.24	0.047

*Note:* Model selection results based on corrected Akaike Information Criterion (AICc) for the variables that influence (a) The overall area of use by boats within the estuary and (b) their utilization distribution (UD) within their local area of use. Predictor variables included: Bathymetry (DEP), distance from the shore (RED), distance from the estuary entrance (OD), and distance from fishing centers (FCD). The table also shows the number of degrees of freedom (df), differences in AICc (ΔAICc), and Akaike weights (ωi).

^a^ This table shows the top‐ranked models from a set of 16 models built using all possible combinations of explanatory variables, in addition to the null model. Models with Δi < 2 are considered equally plausible in explaining the response variables. Bold indicates the most parsimonious model among them (i.e., the best‐supported model with the lowest AICc). Superscripts indicate positive or negative relationships for each variable.

At the scale of area use (third‐order of selection), the best‐fitting model indicated that the utilization distribution (UD) of Guiana dolphins was negatively influenced by UDboats and FCD but positively influenced by DEP and RED (Table 1b). Therefore, Guiana dolphins were more likely to occur in deeper areas that were farther from the river edge and near fishing centers, and had less boat traffic. At this scale, boats were more frequently observed in deeper areas, but unlike the dolphins, they selected areas closer to the river edge and farther from fishing centers. The distance to the ocean (OD) did not influence the UD of either dolphins or boats.

## 4 Discussion

Guiana dolphins and boats in the estuary overlap significantly in their spatial distribution, as both are associated with similar environmental features, particularly deeper areas. However, dolphins occupy a narrower environmental space than boats, indicating greater selectivity in habitat use and suggesting potential spatial constraints linked to disturbance.

Dolphin groups were recorded throughout the estuary but showed a preference for relatively deeper areas and zones farther from the margins within the estuarine system, where the presence of debris and stranded boats is observed along the shores. In addition, dolphins were more frequently recorded near fishing hubs, indicating that their spatial distribution is shaped by the interaction between habitat features and human‐related activities.

The preference for deeper areas within the estuary is consistent with previous studies in Pontal Bay (Santos, Alvarez, et al. 2010; Le Pendu et al. 2024) and other regions, such as Guanabara Bay (Azevedo et al. 2007) and the Cananéia Estuarine‐Lagoon Complex (Ferro de Godoy et al. 2015), which report non‐random habitat use and selection of specific environmental features by Guiana dolphins. Rather than indicating avoidance of shallow habitats per se, this pattern likely reflects fine‐scale habitat selection within a predominantly shallow coastal system.

Previous studies have also shown that Guiana dolphins may adjust their space use in response to anthropogenic pressures, including avoidance of areas with higher levels of human disturbance (Azevedo et al. 2007; Wedekin et al. 2010). In addition, although the use of natural and physical barriers for foraging has been documented for the species (de Meirelles et al. 2023; Pierry et al. 2023), we did not observe spatial patterns consistent with a barrier‐feeding strategy along river edges in Pontal Bay. This may reflect habitat constraints or disturbance associated with these areas, but behavioral observations would be required to confirm this interpretation.

Guiana dolphins showed a negative relationship with distance to fishing centers (FCD) in both second‐ and third‐order selection, indicating higher use of areas closer to these locations. In this study, fishing centers represent landing sites and associated facilities rather than active fishing grounds. This spatial association may reflect enhanced foraging opportunities linked to processes occurring at or near these sites. Interactions between marine mammals and fisheries, including bycatch and depredation, are widespread across both commercial and small‐scale fisheries (Jog et al. 2022), and depredation behavior (where individuals remove captured fish from nets or lines) has been increasingly documented (Read and Ead 2008). Such interactions are facilitated by the opportunistic foraging behavior of cetaceans and their tendency to occupy coastal habitats that overlap with artisanal fishing activities (Marçalo et al. 2024). In this context, fishing centers may act as focal points of fish handling and processing, where organic remains, discards, or bait may become available, potentially attracting dolphins independently of fishing effort at sea. More broadly, interactions between cetaceans and fisheries often arise from spatial overlap between human activities and areas used by dolphins (Seminara et al. 2019). However, as prey availability, fishing practices, and dolphin foraging behavior were not directly quantified, the mechanisms underlying this pattern cannot be confirmed. At finer scales, the negative effect of FCD may also reflect localized disturbance or risk, suggesting a balance between potential energetic benefits with exposure to anthropogenic pressures, as reported in other regions (de Meirelles et al. 2023).

The area used by Guiana dolphin groups in Pontal Bay lies largely within the area used by boats, indicating substantial spatial overlap. This confirms that both dolphins and vessels use areas with similar physical characteristics, such as deeper waters, and highlights the potential risk of negative interactions, including collisions. However, this overlap masks important fine‐scale patterns. Within these shared areas, dolphins more intensely use areas with lower boat density, suggesting localized spatial segregation at short spatial scales, consistent with avoidance behavior (Carrera et al. 2008; Pirotta et al. 2015; New et al. 2020).

The main areas of boat activity were located near the deepest channel in the northern part of the estuary (Figure 4C) and near Cristo Beach. The channel is used mainly by inboard motorboats, as its greater depth accommodates larger vessels, while Cristo Beach attracts a mix of outboard motorboats and non‐motorized boats for recreational purposes. In general, motorized marine traffic is one of the primary sources of disturbance for cetaceans, especially coastal species, as it disrupts their natural behaviors, alters their acoustic environment, and consequently affects their survival and reproductive rates (Azevedo et al. 2007; Marega et al. 2018; Kassamali‐Fox et al. 2020; de Almeida et al. 2024). Among motorized vessels, outboard motorboats may contribute disproportionately to acoustic disturbance due to higher engine power and speeds, which increase noise emissions (Arveson and Vendittis 2000; Erbe 2002). Acoustic impacts have been observed in other odontocetes, including bottlenose dolphins ( *Tursiops truncatus* ) in heavily trafficked waters (Gospic and Picciulin 2015; Pellegrini et al. 2021), suggesting that similar effects may occur in Pontal Bay.

Beyond vessel presence alone, the magnitude of disturbance is also strongly influenced by vessel behavior, which can be as important as, or even more important than, vessel type in determining dolphins' responses. Factors such as speed, approach distance, directionality, and movement predictability are key determinants of disturbance intensity. Fast and unpredictable vessel movements are consistently associated with stronger behavioral responses, including evasive movements, increased swimming speed, and changes in direction, while slower and more predictable vessels often result in weaker or negligible responses (Ng and Leung 2003; Stensland and Berggren 2007; Mills et al. 2023). For example, studies on solitary dolphins have shown that predictable and slow vessel movements may elicit limited observable behavioral changes, even in the presence of repeated interactions (Díaz López et al. 2008). Rapid or direct approaches, in contrast, may limit dolphins' ability to anticipate and avoid vessels, increasing boat disturbance and collision risk. Therefore, understanding not only where and which vessels operate, but also how they operate, is essential to accurately interpret their ecological impacts.

The risk of collision is another concern associated with all motorized boats, and this risk is particularly high for outboard motorboats due to their high speeds, erratic movements, and wide distribution throughout the estuary. Collisions have been documented as a cause of mortality for various dolphin species globally, including Hector's dolphins ( *Cephalorhynchus hectori* ) in New Zealand (Stone and Yoshinaga 2000), Indo‐Pacific humpback dolphins ( *Sousa chinensis* ), and finless porpoises ( *Neophocaena phocaenoides* ) in Hong Kong (Parsons and Jefferson 2000). Furthermore, fractures and trauma in *S. guianensis* resulting from boat collisions have been documented in northern Rio de Janeiro (Van Bressem et al. 2007), the Cananéia estuary (Santos, Campolim, et al. 2010), and Paranaguá Bay (Domiciano et al. 2016).

Although Guiana dolphins reduce their use of areas with intense boat traffic at fine spatial scales, they preferentially select environmental features that spatially coincide with those used by vessels, such as deeper waters and areas associated with fishing activity. In this context, fishing grounds and navigation channels may function as predictable foraging areas, as documented in other systems where cetaceans exploit resources associated with fishing activities (Bearzi 2002; Read and Ead 2008), but at the cost of higher interaction with vessels (de Meirelles et al. 2023). As prey distribution was not assessed in this study, this interpretation remains hypothetical.

At first glance, the contrasting effects of boat traffic across spatial scales may appear contradictory, with dolphins showing a positive association with boat use at the broader (second‐order) scale and a negative association at the finer (third‐order) scale. However, this pattern reflects scale‐dependent habitat selection processes rather than inconsistency.

At the broader scale, both dolphins and boat activity occur in areas characterized by similar environmental features, particularly deeper areas and zones associated with fishing activity, which likely offer enhanced foraging opportunities. As a result, the apparent positive association with boat traffic at this scale is likely indirect, arising from shared habitat preferences rather than a causal attraction to vessels.

In contrast, at finer spatial scales within these shared areas, dolphins adjust their space use in relation to local variation in boat density. This pattern is consistent with localized avoidance, although direct behavioral responses were not assessed. Such scale‐dependent responses are consistent with hierarchical habitat selection, in which different ecological processes operate across spatial scales (Johnson 1980).

This scale‐dependent pattern reflects a trade‐off between access to profitable foraging habitats and exposure to anthropogenic disturbance, where dolphins appear to tolerate boat presence at broader spatial scales while exhibiting localized avoidance at finer scales. This behavioral adjustment is consistent with frameworks describing wildlife responses to human disturbance in terms of tolerance, habituation, and sensitization (Bejder et al. 2009). Similar trade‐offs between energetic gains and risk exposure have been documented across a range of marine and terrestrial systems (Lima and Dill 1990; Brown 1999; Frid and Dill 2002), and in other coastal cetaceans (e.g., Fertl 1997), reinforcing the idea that animals may persist in resource‐rich but risky habitats when the energetic benefits outweigh the costs.

Notably, our analysis was restricted to spatial patterns, which indicate general avoidance of areas with intense boat activity. However, because dolphin and boat trajectories were collected independently and not simultaneously, we were unable to assess their temporal co‐occurrence or direct interactions. As a result, it remains unclear whether dolphins adjust their habitat use in real time in response to boat presence. Future research should therefore incorporate temporal variation to better understand how these trade‐offs are mediated.

Finally, the Guiana dolphin's preference for deeper areas farther from the coast, combined with the silting and narrowing of the estuary, raises concerns about the reduction in available habitat and increasing spatial overlap with boats over time. These processes have been associated with the construction of a cable‐stayed bridge at the mouth of the estuary, which began after the period covered by this study (2017 onwards, Le Pendu et al. 2024). Subsequent research indicates that bridge construction altered local geomorphology, including the expansion of a sandbank, which in turn modified dolphin space use, likely through changes in prey distribution (Le Pendu et al. 2024). As our dataset represents pre‐construction conditions (2015–2016), the patterns described here provide a baseline against which these subsequent changes can be interpreted.

The estuarine system has likely undergone additional anthropogenic changes since then, including increased vessel traffic and ongoing geomorphological alterations. Consequently, the spatial patterns described here should be interpreted as a baseline rather than a current representation of the system.

Nonetheless, the main processes identified, namely the overlap between dolphins and vessels driven by shared environmental preferences, and the fine‐scale spatial adjustments indicative of disturbance avoidance, are likely to remain relevant, as they reflect fundamental ecological mechanisms rather than transient conditions. Therefore, while updated data are needed to assess the current magnitude of interactions, our findings still provide a robust framework for understanding dolphin–vessel dynamics and for informing management strategies in estuarine environments.

From a management perspective, reducing spatial overlap alone may not be feasible, as both dolphins and boats are attracted to the same environmental features. Instead, management actions should focus on regulating vessel behavior within areas of high dolphin use. Measures such as speed restrictions in core foraging zones, spatial or temporal zoning to limit traffic during periods of high dolphin use, and guidelines for vessel approach distances and movement patterns could reduce disturbance while maintaining human use of the estuary. Additionally, cumulative impacts from habitat modification, increasing vessel traffic, and ongoing environmental changes, such as sedimentation and estuarine narrowing, should be considered, as they may progressively reduce habitat availability and intensify human‐wildlife interactions over time. Overall, the spatial distribution of Guiana dolphins in Pontal Bay reflects the combined influence of environmental features and human activities, with fine‐scale spatial adjustments consistent with disturbance avoidance. These findings highlight the importance of integrating habitat protection with the regulation of human activities in estuarine systems. Further research incorporating temporal dynamics and updated data will be essential to assess how ongoing environmental changes may alter these patterns.

## Author Contributions

**Alice Lima:** conceptualization (equal), data curation (equal), visualization (equal), writing – original draft (equal), writing – review and editing (equal). **Gastón Andrés Fernandez Giné:** formal analysis (equal), methodology (equal), validation (equal), visualization (equal), writing – review and editing (equal). **Khamila Tondinelli Souza Cruz:** conceptualization (equal), data curation (equal), formal analysis (equal), funding acquisition (equal), investigation (equal), methodology (equal), writing – original draft (equal). **Niel Nascimento Teixeira:** investigation (equal), methodology (equal), resources (equal). **Gil Marcelo Reuss Strenzel:** formal analysis (equal), investigation (equal), methodology (equal). **Yvonnick Le Pendu:** conceptualization (equal), data curation (equal), funding acquisition (equal), project administration (equal), resources (equal), supervision (equal), validation (equal), writing – review and editing (equal).

## Funding

This work was supported by Universidade Estadual de Santa Cruz, PROBOL Scholarship, Fundação de Amparo à Pesquisa do Estado da Bahia, Project PET0032/2012, Coordenação de Aperfeiçoamento de Pessoal de Nível Superior, Master's degree Scholarship, Animal Behavior Society, Small Grant.

## Conflicts of Interest

The authors declare no conflicts of interest.

## Data Availability

The data and R scripts supporting the results of this study are available in the Dryad Digital Repository. During the peer‐review process, the data and code are accessible to editors and reviewers via the following public link: https://doi.org/10.5061/dryad.tdz08kqdb. Upon acceptance of the manuscript, all data and code will be made publicly available and assigned a permanent DOI.

## References

[ece374004-bib-0001] Araújo, J. P. , A. Souto , L. Geise , and M. E. Araújo . 2008. “The Behavior of *Sotalia guianensis* (Van Beneden) in Pernambuco Coastal Waters, Brazil, and a Further Analysis of Its Reaction to Boat Traffic.” Revista Brasileira de Zoologia 25: 1–9.

[ece374004-bib-0002] Arveson, P. T. , and D. J. Vendittis . 2000. “Radiated Noise Characteristics of a Modern Cargo Ship.” Journal of the Acoustical Society of America 107: 118–129. 10641625 10.1121/1.428344

[ece374004-bib-0003] Azevedo, A. d. F. , A. F. Azevedo , A. M. Oliveira , S. C. Viana , and M. van Sluys . 2007. “Habitat Use by Marine Tucuxis ( *Sotalia guianensis* ) (Cetacea: Delphinidae) in Guanabara Bay, South‐Eastern Brazil.” Journal of the Marine Biological Association of the United Kingdom 87: 201–205. 10.1017/S0025315407054422.

[ece374004-bib-0004] Azevedo, A. d. F. , J. Lailson‐Brito Jr , H. A. Cunha , and M. V. Sluys . 2004. “A Note on Site Fidelity of Marine Tucuxis ( *Sotalia fluviatilis* ) in Guanabara Bay, Southeastern Brazil.” Journal of Cetacean Research and Management 6: 265–268.

[ece374004-bib-0005] Azevedo, A. F. , R. R. Carvalho , M. Kajin , et al. 2017. “The First Confirmed Decline of a Delphinid Population From Brazilian Waters: 2000–2015 Abundance of *Sotalia guianensis* in Guanabara Bay, South‐Eastern Brazil.” Ecological Indicators 79: 1–10. 10.1016/j.ecolind.2017.03.045.

[ece374004-bib-0006] Bartoń, K. 2023. “MuMIn: Multimodal Inference” *R Package Ver. 1.47.5* [Preprint].

[ece374004-bib-0007] Bates, D. , M. Mächler , B. Bolker , and S. Walker . 2015. “Fitting Linear Mixed‐Effects Models Using {lme4}.” Journal of Statistical Software 67: 1–48. 10.18637/jss.v067.i01.

[ece374004-bib-0008] Batista, R. L. G. , M. R. Alvarez , M. do Socorro Santos dos Reis , M. J. Cremer , and A. Schiavetti . 2014. “Site Fidelity and Habitat Use of the Guiana Dolphin, *Sotalia guianensis* (Cetacea: Delphinidae), in the Estuary of the Paraguaçú River, Northeastern Brazil.” North‐Western Journal of Zoology 10: 93–100.

[ece374004-bib-0009] Bearzi, G. 2002. Interactions between Cetaceans and Fisheries in the Mediterranean Sea, Interactions.

[ece374004-bib-0010] Bearzi, G. , S. Agazzi , S. Bonizzoni , M. Costa , and A. Azzellino . 2008. “Dolphins in a Bottle: Abundance, Residency Patterns and Conservation of Bottlenose Dolphins *Tursiops truncatus* in the Semi‐Closed Eutrophic Amvrakikos Gulf, Greece.” East 146: 130–146. 10.1002/aqc.843.

[ece374004-bib-0011] Bejder, L. , A. Samuels , H. Whitehead , H. Finn , and S. Allen . 2009. “Impact Assessment Research: Use and Misuse of Habituation, Sensitisation and Tolerance in Describing Wildlife Responses to Anthropogenic Stimuli.” Marine Ecology Progress Series 395: 177–185. 10.3354/meps07979.

[ece374004-bib-0012] Benhamou, S. 2011. “Dynamic Approach to Space and Habitat Use Based on Biased Random Bridges.” PLoS One 6: e14592. 10.1371/journal.pone.0014592. 21297869 PMC3027622

[ece374004-bib-0013] Benhamou, S. , and D. Cornélis . 2010. “Incorporating Movement Behavior and Barriers to Improve Kernel Home Range Space Use Estimates.” Journal of Wildlife Management 74: 1353–1360. 10.1111/j.1937-2817.2010.tb01257.x.

[ece374004-bib-0014] Bordin, A. , M. Vanhoucke , K. Pineau , et al. 2022. “Study and Conservation of the Guiana Dolphin ( *Sotalia guianensis* ) (Van Bénéden, 1864) in French Guiana.” Latin American Journal of Aquatic Mammals 17: 10–21. 10.5597/lajam00276.

[ece374004-bib-0015] Borobia, M. , S. Siciliano , L. Lodi , and W. Hoek . 1991. “Distribution of the South‐American Dolphin *Sotalia fluviatilis* .” Canadian Journal of Zoology 69: 1025–1039.

[ece374004-bib-0016] Boyd, C. , T. M. Brooks , S. H. M. Butchart , et al. 2008. “Spatial Scale and the Conservation of Threatened Species.” Conservation Letters 1: 37–43. 10.1111/j.1755-263x.2008.00002.x.

[ece374004-bib-0017] BRASIL . 2022. Ministério Do Meio Ambiente. Portaria n°148, de 7 de junho de 2022 Diário Oficial da União ‐ Edição 108 ‐ Seção 1.

[ece374004-bib-0018] Bridge, C. , S. Methion , and B. Díaz López . 2023. “The Impact of Anthropogenic Pollutants on the Distribution of a Marine Top Predator Within a Coastal Estuarine System.” Environmental Monitoring and Assessment 195: 898. 10.1007/s10661-023-11477-2. 37369947

[ece374004-bib-0019] Brown, J. S. 1999. “Vigilance, Patch Use and Habitat Selection: Foraging Under Predation Risk.” Evolutionary Ecology Research 1: 49–71.

[ece374004-bib-0020] Burnham, K. P. , and D. R. Anderson . 2002. Model Selection and Multimodel Inference: A Practical Information‐Theoretic Approach. 2nd editio ed. Springer‐Verlag.

[ece374004-bib-0021] Calenge, C. 2024. “adehabitatHR: Home Range Estimation.” R package version 0.4.22, https://github.com/clementcalenge/adehabitathr.

[ece374004-bib-0022] Carrera, M. L. , E. G. P. Favaro , and A. Souto . 2008. “The Response of Marine Tucuxis ( *Sotalia fluviatilis* ) Towards Tourist Boats Involves Avoidance Behaviour and a Reduction in Foraging.” Animal Welfare 17: 117–123.

[ece374004-bib-0023] da Silva, J. M. S. , A. P. M. Mariano , and M. F. Silva . 2019. “Combination of Factors That Increase the Risk of Contamination by Geohelminths Larvae in the South Coast of Bahia, Brazil.” Brazilian Journal of Development 5: 29254–29270. 10.34117/bjdv5n12-084.

[ece374004-bib-0024] da Silva, V. M. F. , and R. C. Best . 1996. “ *Sotalia fluviatilis* .” Mammalian Species 527: 1–7.

[ece374004-bib-0025] Daura‐Jorge, F. G. , M. R. Rossi‐Santos , L. L. Wedekin , and P. C. Simões‐Lopes . 2007. “Behavioral Patterns and Movement Intensity of *Sotalia guianensis* (P.J. van Bénéden) (Cetacea, Delphinidae) in Two Differente Areas on the Brazilian Coast.” Revista Brasileira de Zoologia 24: 265–270.

[ece374004-bib-0026] David, L. 2002. Cetaceans of the Mediterranean and Black Seas State. Cetaceans of the Mediterranean and Black Seas.

[ece374004-bib-0027] de Almeida, I. G. , A. R. Percequillo , and M. M. Rollo . 2024. “Surviving the Tide: Assessing Guiana Dolphin Persistence Amidst Growing Threats in a Protected Estuary in South‐Eastern Brazil.” Journal for Nature Conservation 82: 126713. 10.1016/j.jnc.2024.126713.

[ece374004-bib-0028] de Jesus Lobo, A. , L. L. Wedekin , T. Sobral‐Souza , and Y. Le Pendu . 2021. “Potential Distribution of Guiana Dolphin ( *Sotalia guianensis* ): A Coastal‐Estuarine and Tropical Habitat Specialist.” Journal of Mammalogy 102: 308–318. 10.1093/jmammal/gyaa153.

[ece374004-bib-0029] de Meirelles, A. , K. F. Choi‐Lima , T. M. Campos , E. L. de Araújo Monteiro‐Filho , and T. M. da Cruz Lotufo . 2023. “Habitat Use of Guiana Dolphin ( *Sotalia guianensis* ) in a Heavily Urbanized Embayment.” Marine Ecology Progress Series 722: 195–206. 10.3354/meps14430.

[ece374004-bib-0030] Diáz López, B. 2019. “‘Hot Deals at Sea’: Responses of a Top Predator (Bottlenose Dolphin, *Tursiops truncatus* ) to Human‐Induced Changes in the Coastal Ecosystem.” Behavioral Ecology 30: 291–300. 10.1093/beheco/ary162.

[ece374004-bib-0031] Díaz López, B. , and S. Methion . 2017. “The Impact of Shellfish Farming on Common Bottlenose Dolphins' Use of Habitat: Running Head: Impact of Mussel Farming on Bottlenose Dolphins.” Marine Biology 164: 0. 10.1007/s00227-017-3125-x.

[ece374004-bib-0032] Díaz López, B. , J. A. B. Shirai , A. B. Prieto , and P. M. Fernández . 2008. “Diving Activity of a Solitary Wild Free Ranging Bottlenose Dolphin ( *Tursiops truncatus* ).” Journal of the Marine Biological Association of the United Kingdom 88: 1153–1157. 10.1017/S0025315408000921.

[ece374004-bib-0033] Doledec, S. , D. Chessel , and C. Gimaret‐Carpentier . 2000. “Niche Separation in Community Analysis: A New Method.” Ecology 81: 2914. 10.2307/177351.

[ece374004-bib-0034] Domiciano, I. G. , C. Domit , M. K. Broadhurst , M. S. Koch , and A. P. F. Bracarense . 2016. “Assessing Disease and Mortality Among Small Cetaceans Stranded at a World Heritage Site in Southern Brazil.” PLoS One 11: e0149295. 10.1371/journal.pone.0149295. 26871703 PMC4752507

[ece374004-bib-0035] Erbe, C. 2002. “Underwater Noise of Whale‐Watching Boats and Potential Effects on Killer Whales ( *Orcinus orca* ), Based on an Acoustic Impact Model.” Marine Mammal Science 18: 394–418. 10.1111/j.1748-7692.2002.tb01045.x.

[ece374004-bib-0036] Erbe, C. , S. A. Marley , R. P. Schoeman , J. N. Smith , L. E. Trigg , and C. B. Embling . 2019. “The Effects of Ship Noise on Marine Mammals—A Review.” Frontiers in Marine Science 6: 606. 10.3389/fmars.2019.00606.

[ece374004-bib-0037] FAO . 2011. Coastal Fisheries of Latin America and the Caribbean, edited by S. Salas , R. Chuenpagdee , A. Charles , and J. C. Seijo . Food and Agriculture Organization of the United Nations (FAO Fisheries and Aquaculture Technical Paper No. 544).

[ece374004-bib-0038] Ferro de Godoy, D. , A. Andriolo , and G. de Fatima Filla . 2015. “The Influence of Environmental Variables on Estuarine Dolphins ( *Sotalia guianensis* ) Spatial Distribution and Habitat Used in the Estuarine Lagunar Complex of Cananéia, Southeastern Brazil.” Ocean and Coastal Management 106: 68–76. 10.1016/j.ocecoaman.2015.01.013.

[ece374004-bib-0039] Fertl, D. 1997. “Cetacean Interactions With Trawls : A Preliminary Review.” Journal of Northwest Atlantic Fishery Science 22: 219–248.

[ece374004-bib-0040] Flores, P. A. C. , V. M. F. da Silva , and D. d. C. Fettuccia . 2018. “Tucuxi and Guiana Dolphins.” In Encyclopedia of Marine Mammals, 1024–1027. Elsevier. 10.1016/B978-0-12-804327-1.00264-8.

[ece374004-bib-0041] Frid, A. , and L. Dill . 2002. “Human‐Caused Disturbance Stimuli as a Form of Predation Risk.” Ecology and Society 6: art11. 10.5751/es-00404-060111.

[ece374004-bib-0042] Geise, L. , N. Gomes , and R. Cerqueira . 1999. “Behaviour, Habitat Use and Population Size of *Sotalia fluviatilis* (Gervais, 1853) (Cetacea, Delphinidae) in the Cananéia Estuary Region, São Paulo, Brazil.” Revista Brasileira de Biologia 59: 183–194.

[ece374004-bib-0043] Gospic, N. R. , and M. Picciulin . 2015. “Changes in Whistle Structure of Resident Bottlenose Dolphins in Relation to Underwater Noise and Boat Traffic.” Marine Pollution Bulletin 105: 193–198. 10.1016/j.marpolbul.2016.02.030. 26917094

[ece374004-bib-0044] Halpern, B. S. , S. Walbridge , K. A. Selkoe , et al. 2008. “A Global Map of Human Impact on Marine Ecosystems.” Science 319: 948–952. 10.1126/science.1149345. 18276889

[ece374004-bib-0045] Hijmans, R. 2023. “_raster: Geographic Data Analysis and Modeling_.” R package version 3.6–26.

[ece374004-bib-0046] Irvine, A. B. , M. Scott , R. S. Wells , and J. H. Kaufmann . 1981. “Movements and Activities of the Atlantic Bottlenose Dolphin, *Tursiops truncatus* , Near Sarasota, Florida.” Fishery Bulletin 79: 671–688.

[ece374004-bib-0047] Jensen, F. , L. Bejder , M. Wahlberg , N. Aguilar de Soto , M. Johnson , and P. T. Madsen . 2009. “Vessel noise effects on delphinid communication.” Marine Ecology Progress Series 395: 161–175. 10.3354/meps08204.

[ece374004-bib-0048] Jog, K. , D. Sutaria , A. Diedrich , A. Grech , and H. Marsh . 2022. “Marine Mammal Interactions With Fisheries: Review of Research and Management Trends Across Commercial and Small‐Scale Fisheries.” Frontiers in Marine Science 9: 758013. 10.3389/fmars.2022.758013.

[ece374004-bib-0049] Johnson, D. H. 1980. “The Comparison of Usage and Availability Measurements for Evaluating Resource Preference.” Ecology 61: 65–71.

[ece374004-bib-0050] Kassamali‐Fox, A. , F. Christiansen , L. J. May‐Collado , E. A. Ramos , and B. A. Kaplin . 2020. “Tour Boats Affect the Activity Patterns of Bottlenose Dolphins ( *Tursiops truncatus* ) in Bocas del Toro, Panama.” PeerJ 8: 1–22. 10.7717/peerj.8804. PMC7115753 32266117

[ece374004-bib-0051] La Manna, G. , F. Ronchetti , F. Perretti , and G. Ceccherelli , 2023. “Areas of Spatial Overlap Between Common Bottlenose Dolphin, Recreational Boating, and Small‐Scale Fishery: Management Insights From Modelling Exercises.” PeerJ 11: 1–25. 10.7717/peerj.16111. PMC10542390 37790616

[ece374004-bib-0052] Le Pendu, Y. , A. Lima , E. Gomes , W. Silva , K. T. S. Cruz , and G. A. F. Giné . 2024. “Response of Guiana Dolphins to the Construction of a Bridge in Ilhéus, Northeastern Brazil.” PLoS One 19: 1–15. 10.1371/journal.pone.0312476. PMC11567520 39546441

[ece374004-bib-0053] Lima, S. L. , and L. M. Dill . 1990. “Behavioral Decisions Made Under the Risk of Predation: A Review and Prospectus.” Canadian Journal of Zoology 68: 619–640.

[ece374004-bib-0054] Lipsey, M. K. , D. E. Naugle , J. Nowak , and P. M. Lukacs . 2017. “Extending Utility of Hierarchical Models to Multi‐Scale Habitat Selection.” Diversity and Distributions 23: 783–793. 10.1111/ddi.12567.

[ece374004-bib-0055] Lotze, H. K. , H. S. Lenihan , B. J. Bourque , et al. 2006. “Depletion Degradation, and Recovery Potential of Estuaries and Coastal Seas.” Science 312: 1806–1809. 10.1126/science.1128035. 16794081

[ece374004-bib-0056] MacLeod, C. D. 2009. “Global Climate Change, Range Changes and Potential Implications for the Conservation of Marine Cetaceans: A Review and Synthesis.” Endangered Species Research 7: 125–136. 10.3354/esr00197.

[ece374004-bib-0057] Mann, K. H. 2000. Ecology of Coastal Waters, edited by K. H. Mann , Second Edi ed. Blackwell Science.

[ece374004-bib-0058] Marçalo, A. , V. Samel , F. Carvalho , M. Frade , K. Erzini , and J. M. S. Gonçalves . 2024. “Evaluating Dolphin Interactions With Bottom‐Set Net Fisheries Off Southern Iberian Atlantic Waters.” Fisheries Research 278: 107100. 10.1016/j.fishres.2024.107100.

[ece374004-bib-0059] Marega, M. , G. Henrique , Y. le Pendu , P. Silva , and A. Schiavetti . 2018. “Behavioral Responses of *Sotalia guianensis* (Cetartiodactyla, Delphinidae) to Boat Approaches in Northeast Brazil.” Latin American Journal of Aquatic Research 46: 268–279. 10.3856/vol46-issue2-fulltext-3.

[ece374004-bib-0062] Markowitz, T. M. , A. D. Harlin , B. Würsig , and C. J. Mcfadden , 2004. “Dusky Dolphin Foraging Habitat: Overlap With Aquaculture in New Zealand.” 149: 133–149. 10.1002/aqc.602.

[ece374004-bib-0060] Matos, I. d. S. 2017. Caracterização morfossedimentar do fundo estuarino da Baía do Pontal em Ilhéus‐Ba. Universidade Federal de Santa Catarina.

[ece374004-bib-0061] Maxwell, S. M. , E. L. Hazen , R. L. Lewison , et al. 2015. “Dynamic Ocean Management: Defining and Conceptualizing Real‐Time Management of the Ocean.” Marine Policy 58: 42–50. 10.1016/j.marpol.2015.03.014.

[ece374004-bib-0063] Mendes, S. , W. Turrell , T. Lütkebohle , and P. Thompson . 2002. “Influence of the Tidal Cycle and a Tidal Intrusion Front on the Spatio‐Temporal Distribution of Coastal Bottlenose Dolphins.” Marine Ecology Progress Series 239: 221–229.

[ece374004-bib-0064] Methion, S. , and B. Díaz López . 2019. “Natural and Anthropogenic Drivers of Foraging Behaviour in Bottlenose Dolphins: Influence of Shellfish Aquaculture.” Aquatic Conservation: Marine and Freshwater Ecosystems 29: 927–937. 10.1002/aqc.3116.

[ece374004-bib-0065] Mills, E. M. M. , S. Piwetz , and D. N. Orbach . 2023. “Vessels Disturb Bottlenose Dolphin Behavior and Movement in an Active Ship Channel.” Animals 13: 1–18. 10.3390/ani13223441 PMC10668690 38003059

[ece374004-bib-0066] Nabe‐Nielsen, J. , F. M. Beest , V. Grimm , R. M. Sibly , J. Teilmann , and P. M. Thompson . 2018. “Predicting the Impacts of Anthropogenic Disturbances on Marine Populations.” Conservation Letters 11: 1–8. 10.1111/conl.12563.

[ece374004-bib-0067] New, L. , D. Lusseau , and R. Harcourt . 2020. “Dolphins and Boats: When Is a Disturbance, Disturbing?” Frontiers in Marine Science 7: 1–13. 10.3389/fmars.2020.00353.32802822

[ece374004-bib-0068] Ng, S. L. , and S. Leung . 2003. “Behavioral Response of Indo‐Pacific Humpback Dolphin ( *Sousa chinensis* ) to Vessel Traffic.” Marine Environmental Research 56: 555–567. 10.1016/S0141-1136(03)00041-2. 12927738

[ece374004-bib-0069] Nowacek, D. P. , L. E. S. L. E. Y. H. Thorne , D. A. V. I. D. W. Johnston , and P. E. T. E. R. L. Tyack . 2007. “Responses of Cetaceans to Anthropogenic Noise.” Mammal Review 37: 81–115. 10.1111/j.1365-2907.2007.00104.x.

[ece374004-bib-0070] Paitach, R. L. , P. C. Simões‐Lopes , and M. J. Cremer . 2017. “Tidal and Seasonal Influences in Dolphin Habitat Use in a Southern Brazilian Estuary.” Scientia Marina 81: 49–56. 10.3989/scimar.04495.25a.

[ece374004-bib-0071] Parsons, E. C. M. , and T. A. Jefferson . 2000. “Post‐Mortem Investigations on Stranded Dolphins and Porpoises From Hong Kong Waters.” Journal of Wildlife Diseases 36: 342–356. 10.7589/0090-3558-36.2.342. 10813617

[ece374004-bib-0072] Pearson, H. C. , R. L. Vaughn‐Hirshorn , M. Srinivasan , and B. Würsig . 2012. “Avoidance of Mussel Farms by Dusky Dolphins ( *Lagenorhynchus obscurus* ) in New Zealand.” New Zealand Journal of Marine and Freshwater Research 46: 567–574. 10.1080/00288330.2012.712977.

[ece374004-bib-0073] Pellegrini, A. Y. , B. Romeu , S. N. Ingram , and F. G. Daura‐Jorge . 2021. “Boat Disturbance Affects the Acoustic Behaviour of Dolphins Engaged in a Rare Foraging Cooperation With Fishers.” Animal Conservation 24: 613–625. 10.1111/acv.12667.

[ece374004-bib-0074] Pierry, J. C. , M. E. Morete , E. L. A. Monteiro Filho , and C. R. Teixeira . 2023. “Guiana Dolphins Use Mangrove Margins as a Natural Barrier to Chase Fish Prey.” Ethology 130: e13411. 10.1111/eth.13411.

[ece374004-bib-0075] Pinheiro, J. , and D. Bates . 2023. “nlme: Linear and Nonlinear Mixed Effects Models.” R package version 3. Org, 1–164. https://cran.r‐project.org/web/packages/nlme/nlme.pdf.

[ece374004-bib-0076] Pinheiro, J. C. , and D. M. Bates . 2000. Mixed‐Effects Models in S and S‐PLUS. Springer‐Verlag (Statistics and Computing). 10.1007/b98882.

[ece374004-bib-0077] Pirotta, E. , N. D. Merchant , P. M. Thompson , T. R. Barton , and D. Lusseau . 2015. “Quantifying the Effect of Boat Disturbance on Bottlenose Dolphin Foraging Activity.” Biological Conservation 181: 82–89. 10.1016/j.biocon.2014.11.003.

[ece374004-bib-0078] QGIS.org . 2026. QGIS Geographic Information System. QGIS Association. http://www.qgis.org.

[ece374004-bib-0079] R Core Team . 2024. R: A Language and Environment for Statistical Computing. R Foundation for Statistical Computing.

[ece374004-bib-0080] Read, A. J. , P. Drinker , and S. Northridge . 2006. “Bycatch of Marine Mammals in U.S. and Global Fisheries.” Conservation Biology 20: 163–169. 10.1111/j.1523-1739.2006.00338.x. 16909669

[ece374004-bib-0081] Read, A. J. , and A. N. J. R. Ead . 2008. “The Looming Crisis: Interactions Between Marine Mammals and Fisheries.” Journal of Mammalogy 89: 541–548.

[ece374004-bib-0082] Redfern, J. V. , M. C. Ferguson , E. A. Becker , et al. 2006. “Techniques for Cetacean – Habitat Modeling.” Marine Ecology Progress Series 310: 271–295.

[ece374004-bib-0084] Ribeiro, S. , F. A. Viddi , J. L. Cordeiro , and T. R. O. Freitas . 2007. “Fine‐Scale Habitat Selection of Chilean Dolphins ( *Cephalorhynchus eutropia* ): Interactions With Aquaculture Activities in Southern Chiloé Island, Chile.” Journal of the Marine Biological Association of the United Kingdom 87: 119–128. 10.1017/S0025315407051594.

[ece374004-bib-0085] Rossi‐santos, M. R. , L. L. Wedekin , and E. L. A. . de A Monteiro‐Filho . 2007. “Residence and Site Fidelity of *Sotalia guianensis* in Caravelas River Estuary, Eastern Brazil.” Journal of the Marine Biological Association of the United Kingdom 87: 207–212. 10.1017/S0025315407055683.

[ece374004-bib-0086] Santos, M. C. O. , M. B. Campolim , I. S. Parada , P. Dunker , and E. Silva . 2010. “The Triumph of the Commons: Working Towards the Conservation of Guiana Dolphins ( *Sotalia guianensis* ) in the Cananéia Estuary, Brazil.” Latin American Journal of Aquatic Mammals 8: 187–190. 10.5597/lajam00169.

[ece374004-bib-0087] Santos, M. C. O. , S. Rosso , R. A. dos Santos , S. H. B. Lucato , and M. Bassoi . 2002. “Insights on Small Cetacean Feeding Habits in Southeastern Brazil.” Aquatic Mammals 28: 38–45.

[ece374004-bib-0088] Santos, U. A. D. , M. R. Alvarez , A. C. Schilling , G. M. R. Strenzel , and Y. L. Pendu . 2010. “Spatial Distribution and Activities of the Estuarine Dolphin *Sotalia guianensis* (van Bénédén, 1864) (Cetacea, Delphinidae) in Pontal Bay, Ilhéus, Bahia, Brazil.” Biota Neotropica 10: 1–7. 10.1590/S1676-06032010000200007.

[ece374004-bib-0089] Secchi, E. , M. C. d. O. Santos , and R. Reeves . 2018. “*Sotalia guianensis* (errata version published in 2019).” 10.2305/IUCN.UK.2018-2.RLTS.T181359A144232542.en. Accessed on 07 July 2026. The IUCN Red List of Threatened Species 2018: e.T181359A144232542.

[ece374004-bib-0090] Seminara, C. I. , M. L. V. Barbosa‐Filho , and Y. Le Pendu . 2019. “Interactions Between Cetaceans and Artisanal Fishermen From Ilhéus, Bahia—Brazil.” Biota Neotropica 19: e20190742.

[ece374004-bib-0091] Silva, I. M. , N. Jesus , J. Castro , and A. R. Luís . 2024. “The Effects of Vessel Traffic on the Behavior Patterns of Common Dolphins in the Tagus Estuary (Portugal).” Animals 14: 1–12. 10.3390/ani14202998. PMC11506354 39457928

[ece374004-bib-0092] Stensland, E. , and P. Berggren . 2007. “Behavioural Changes in Female Indo‐Pacific Bottlenose Dolphins in Response to Boat‐Based Tourism.” Marine Ecology Progress Series 332: 225–234. 10.3354/meps332225.

[ece374004-bib-0093] Stone, G. S. , and A. Yoshinaga . 2000. “Hector's Dolphin *Cephalorhynchus hectori* Calf Mortalities May Indicate New Risks From Boat Traffic and Habituation.” Pacific Conservation Biology 6: 167–170.

[ece374004-bib-0094] Tardin, R. , G. Maricato , J. J. Kiszka , et al. 2025. “Optimistic Climate Mitigation Scenario Halves Projected Range Loss in a Neotropical Dolphin.” Ocean and Coastal Management 269: 107800. 10.1016/j.ocecoaman.2025.107800.

[ece374004-bib-0095] Tardin, R. H. , I. S. Maciel , M. A. Espécie , G. Melo‐Santos , S. M. Simão , and M. A. S. Alves . 2020. “Modelling Habitat Use by the Guiana Dolphin, *Sotalia guianensis* , in South‐Eastern Brazil: Effects of Environmental and Anthropogenic Variables, and the Adequacy of Current Management Measures.” Aquatic Conservation: Marine and Freshwater Ecosystems 30: 775–786. 10.1002/aqc.3290.

[ece374004-bib-0096] Tosi, C. H. , and R. G. Ferreira . 2009. “Behavior of Estuarine Dolphin, *Sotalia guianensis* (Cetacea, Delphinidae), in Controlled Boat Traffic Situation at Southern Coast of Rio Grande Do Norte, Brazil.” Biodiversity and Conservation 18: 67–78. 10.1007/s10531-008-9435-z.

[ece374004-bib-0097] Van Bressem, M.‐F. , K. Van Waerebeek , J. C. Reyes , et al. 2007. “A Preliminary Overview of Skin and Skeletal Diseases and Traumata in Small Cetaceans From South American Waters.” Latin American Journal of Aquatic Mammals 6: 7–42.

[ece374004-bib-0098] Villero, D. , M. Pla , D. Camps , J. Ruiz‐Olmo , and L. Brotons . 2017. “Integrating Species Distribution Modelling Into Decision‐Making to Inform Conservation Actions.” Biodiversity and Conservation 26: 251–271. 10.1007/s10531-016-1243-2.

[ece374004-bib-0099] Wedekin, L. L. , F. G. Daura‐Jorge , V. Q. Piacentini , and P. C. Simões‐Lopes . 2007. “Seasonal Variations in Spatial Usage by the Estuarine Dolphin, *Sotalia guianensis* (van Bénéden, 1864) (Cetacea; Delphinidae) at Its Southern Limit of Distribution.” Brazilian Journal of Biology 67: 1–8. 10.1590/s1519-69842007000100002 17505744

[ece374004-bib-0100] Wedekin, L. L. , F. G. Daura‐Jorge , and P. C. Simões‐Lopes . 2010. “Habitat Preferences of Guiana Dolphins, *Sotalia guianensis* (Cetacea: Delphinidae), in Norte Bay, Southern Brazil.” Journal of the Marine Biological Association of the United Kingdom 90: 1561–1570. 10.1017/S0025315410001414.

